# Supplementary material for: The interactome of intact mitochondria by cross-linking mass spectrometry provides evidence for coexisting respiratory supercomplexes
Source: Mol Cell Proteomics. 2017 Dec 8;17(2):216–32. doi: 10.1074/mcp.RA117.000470 (PMC5795388; doi:10.1074/mcp.RA117.000470)
Supplement: Supplemental Data [file supp_RA117.000470_133922_0_supp_23978_fzffwf.zip › spectra_annotation/mito_spectra_annotation/487-1-12-1-6-1.pdf]

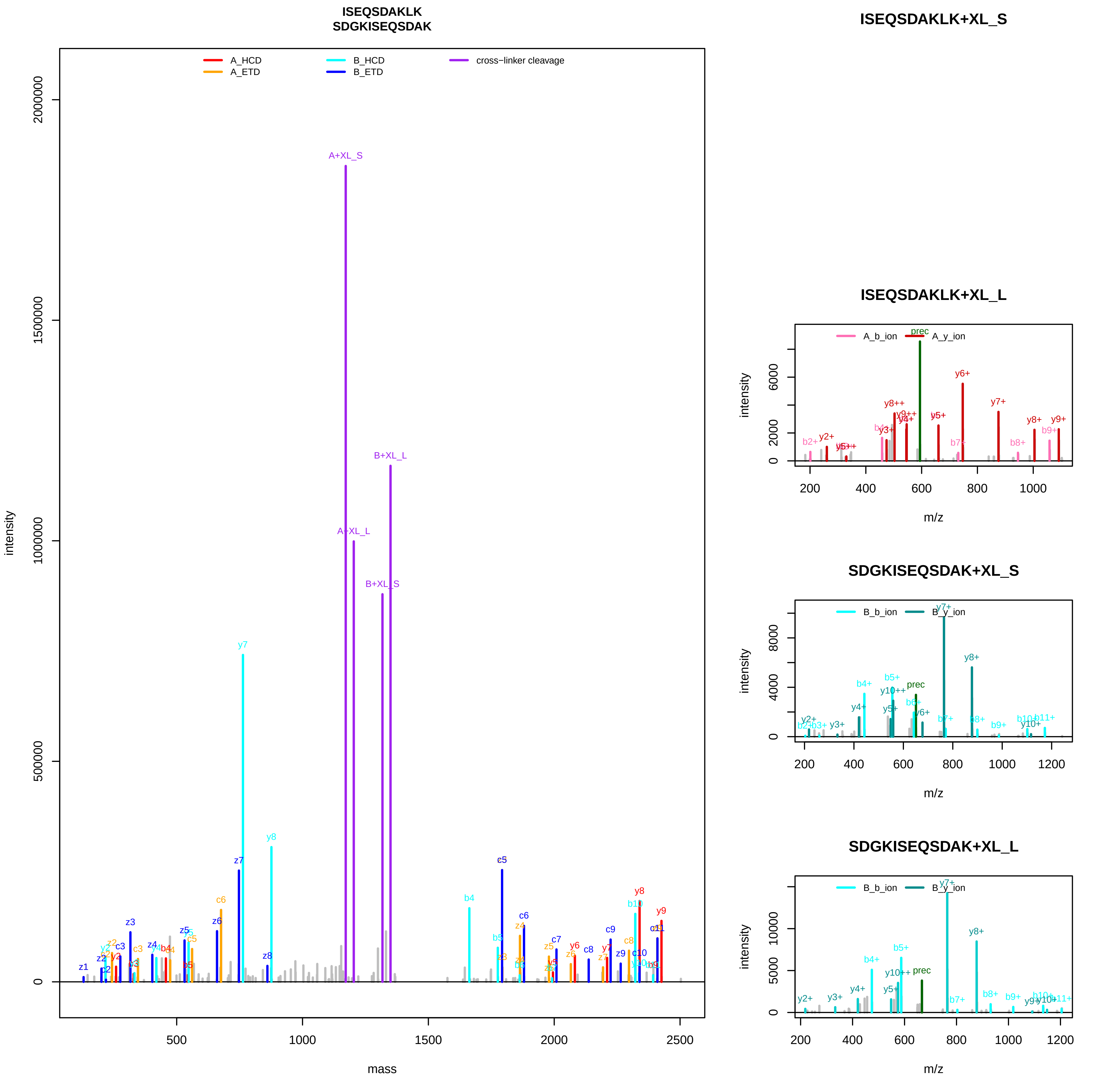
ISEQSDAKLK
SDGKISEQSDAK
A_HCD
A_ETD
B_HCD
B_ETD
cross−linker cleavage
A+XL_S
B+XL_L
A+XL_L
B+XL_S
y7
y8
z7
c5
b4
c6
y8
y9
b10
c11
intensity
mass
0
500000
1000000
1500000
2000000
500
1000
1500
2000
2500
ISEQSDAKLK+XL_S
ISEQSDAKLK+XL_L
A_b_ion
A_y_ion
prec
y6+
y7+
y8++
y8+
y9+
intensity
m/z
0
2000
6000
200
400
600
800
1000
SDGKISEQSDAK+XL_S
B_b_ion
B_y_ion
y7+
y8+
prec
intensity
m/z
0
4000
8000
200
400
600
800
1000
1200
SDGKISEQSDAK+XL_L
B_b_ion
B_y_ion
y7+
y8+
b5+
b4+
prec
intensity
m/z
0
5000
10000
200
400
600
800
1000
1200
